# Association between medication adherence and health-related quality of life in patients with chronic obstructive pulmonary disease

**DOI:** 10.1186/s40780-021-00222-x

**Published:** 2021-11-15

**Authors:** Boyuk Moradkhani, Samaneh Mollazadeh, Parastoo Niloofar, Afsaneh Bashiri, Mohammad Bagher Oghazian

**Affiliations:** 1 Clinical Research Development Unit, Imam Hasan Hospital, North Khorasan University of Medical Sciences, Bojnurd, Iran; 2 Natural Products and Medicinal Plants Research Center, North Khorasan University of Medical Sciences, Bojnurd, Iran; 3 Department of Internal Medicine, Faculty of Medicine, North Khorasan University of Medical Sciences, Bojnurd, Iran

**Keywords:** Chronic obstructive pulmonary disease, Disease severity, Health status, Quality of life, Medication adherence

## Abstract

**Background:**

Chronic Obstructive Pulmonary Disease (COPD) is one of the prominent cause of mortality worldwide. Nowadays, the level of medication adherence in COPD patients is very low, which reduces the clinical therapeutic effects. The purpose of the present study is to investigate the relationship between medication adherence and Health-Related Quality of Life (HRQoL) in COPD patients referred to the pulmonologist’s office.

**Methods:**

This observational study was performed on 100 COPD outpatient cases. Each patient was interviewed to answer questionnaires regarding demographic and clinical information. To assess quality of life, health status, and severity of dyspnea, the St George’s Respiratory Questionnaire - COPD-Specific Version (SGRQ-C), COPD Assessment Test (CAT), and Modified British Medical Research Council (mMRC) questionnaires were used, respectively. Persian version of the Morisky Medication Adherence Scale (MMAS-8-Item) was used to measure medication adherence. To determine the adherence predictors, an ordinal logistic regression analysis was performed.

**Results:**

Out of 100 patients with mean (±SD) age of 61.35 (±10.79) years, 74% had medium and high medication adherence. In the final ordinal logistic model, quality of life, health status, and education level found to have positive effect on medication adherence while polypharmacy had negative effect. We did not find any significant association between age, gender, Body Mass Index (BMI), and other variables with medication adherence.

**Conclusions:**

Patients with high quality of life are more adherent to their medications. Furthermore, patients who have polypharmacy, tend to have less adherence to their medications.

## Background

Chronic Obstructive Pulmonary Disease (COPD) can negatively affect everyday life of patients leading to increased health care costs and long-term adverse effects on health status. Pharmacological treatment of patients with COPD can relieve symptoms, improve lung function, and reduce the exacerbation risk. The most frequent medications to control COPD include short/ long -acting bronchodilators, inhaled corticoids, or a combination of them [1, 2]. Discontinuation of treatment in COPD patients may increase disease severity, hospitalization, and mortality rate [3]. Adherence to inhalants is a complex process affected by a variety of variables including patient-related factors (e.g. age, gender, and comorbidity), different types of treatment regimens (e.g. polypharmacy, dose frequency, and inhalant type), and the interaction between patients and health care providers [4]. Adherence to medication refers to the extent to which the subject complies with the health care provider’s instructions regarding the timing, dose, and duration of the drug being administered [5]. Findings of a systematic review show that non-adherence is common in COPD patients and may cause reduction in therapeutic effects [5]. Nowadays, the degree of adherence to medications in COPD subjects barely exceeds 60% [6]. Despite the promising benefits, the interplay between progressive nature of COPD and the negative effects of poor compliance are not well understood [7]. The most important predictors of poor compliance are low expectations of treatment, comorbidities, depression, aging, smoking and lack of confidence in subjects [7].

Health-Related Quality of Life (HRQoL) is one of the most important factor describing subjects’ health and well-being which is significantly affected by COPD. HRQoL can be regarded as an indicator of successful treatment, when the therapeutic effects are achieved by adherence to medications [5, 8]. Although various aspects of medication adherence and quality of life have been studied in COPD patients, little is known about the relationship between medication adherence and HRQoL [3]. Correspondingly, available studies did not provide any conclusive information about this issue. Additionally, there may be a mutual relation between these two factors. Therefore, medication adherence can be triggered by HRQoL or vice versa [8].

The aim of this study is to identify the association between medication adherence and HRQoL and to assess clinical factors affecting medication adherence in COPD patients.

## Methods

### Study design and population

This is an observational cross-sectional study conducted between September 2019 and March 2020 in the pulmonologist office, which was based on the guidelines of the Helsinki. The research is carried out on COPD patients undergoing a long-term medication treatment. According to Pearson’s correlation coefficient formula, a total number of 100 COPD patients has been selected for the study. All participants have read and signed the informed consent form. The inclusion criteria are 1) developed COPD at least for a year according to spirometric criteria of the GOLD guideline; 2) regular visits to the pulmonologist office every 3 months; 3) under medication treatment for COPD for at least 1 year; 4) no changes in COPD medication during the last 3 months; 5) no serious comorbidities (such as heart, liver, or kidney failures); 6) no definite psychological problems.

### Demographic and clinical factors

Each subject was interviewed to answer a questionnaire about their demographic and clinical factors such as age, gender, weight, height, education level, clinical background, duration of COPD disease, number of exacerbations, duration and number of hospitalization, other chronic underlying diseases, history of vaccination against influenza and pneumococcus, previous and current exposure to cigarettes/opium/livestock farms/cooking ovens/farmlands and the current and previous pulmonary medications. Then, the patients’ medical history was completed according to their medical records. Polypharmacy was defined as regular use of five or more medications daily. Instructions on how to use inhaler devices were handled by a trained nurse in the physician’s office, at each visit.

### Outcome measures

Medication adherence was measured using the Persian version of the eight-item Morisky Medication Adherence Scale (MMAS-8-Item) [9]. This version’s permission was granted to us by Negarandeh et al. [10]. Total obtained scores on the MMAS-8 range from 0 to 8 resulting from the sum of all answer points, and have been categorized into high adherence (score = 8), medium adherence (6 ≤ score < 8), and low adherence (score < 6). The St George’s Respiratory Questionnaire - COPD-Specific Version (SGRQ-C) was used to measure the HRQoL. The responses were in three dimensions as follows; symptoms, level of physical function, and the disease-related psychological effects. The total mean SGRQ-C score reflects the effect of chronic airflow limitation on the general health status. Subjects scoring 25 and higher were classified as having poor quality of life. Modified British Medical Research Council (mMRC) and COPD Assessment Test (CAT) instruments were applied to measure breathlessness and health status impairment, respectively [11]. The mMRC scale consists of five descriptive items to estimate the severity of dyspnea in subjects, in which grade ≥ 2 shows not only greater severity of dyspnea but also poor HRQoL [12]. The CAT test consists of 8 sections including shortness of breath, cough, sputum, sleep status, daily activity, energy levels, anxiety, and chest pain. The score for so-called sections ranges from 0 to 5 in which higher scores (≥10) represent more severe impact of COPD on health status [13]. Each patient was interviewed to fulfill MMAS-8, SGRQ-C, mMRC, and CAT questionnaries after receiving instruction on how to use inhaler devices. Besides, to assess functional severity of the disease the forced expiratory volume in the first second (FEV_1_) was also measured in all cases.

### Statistical analysis

All data were analyzed using SPSS 20.0 software. Descriptive statistics used to compare demographic characteristics and clinical data. Then, the normality of the variables was checked. To test the association between categorical and continuous variables with medication adherence, Pearson’s χ^2^ test or Fisher’s exact test and ANOVA or Kruskal-Wallis were used, respectively. To explore the predictor variables of medication adherence, ordinal logistic regression model was applied considering categorical form of medication adherence (i.e. high, medium, low) as the dependent variable. Statistical significance level was 0.05.

## Results

Overall 100 subjects with mean age (±SD) of 61.35(±10.79) were recruited in this study of whom 51% were male. Also, 91.8% of all female participants were housewives. About 40% of all subjects lived in rural area, 67% were opium abusers, and 24% had history of smoking. Regarding antibiotic consumption, no antibiotics were prescribed for 76% of subjects during the last 2 weeks. Furthermore, 50% of subjects received annual seasonal flu vaccine and 2% of subjects vaccinated against pneumococcal infections. Moreover, 73% of subjects had no history of hospitalization during last year. In terms of the number of years diagnosed with COPD, patients who were diagnosed with the disease between 1 and 2 years ago had the most frequency (20%). Mean FEV_1_% (±SD) for all patients were 49.34(±16.91). Medication adherence was low for 26%, medium for 51% and high for 23% of participants with median (25–75 percentile) of 6.5 (5.75–7.5). Detailed demographic and clinical characteristics of patients are presented in Table 1.

**Table 1:** Demographic data and clinical characteristics of COPD patients

		MMAS-8-Item Categories			
		Low (MMAS < 6)	Medium (6 ≤ MMAS < 8)	High (MMAS = 8)	*P*-Value
Gender	female	16 (61.54%)	20 (39.22%)	13 (56.52%)	0.128
	male	10 (38.46%)	31 (60.78%)	10 (43.48%)	
Age	30–50	2 (7.69%)	6 (11.76%)	7 (30.43%)	0.096
	51–70	16 (61.54%)	35 (68.63%)	15 (65.22%)	
	71–90	8 (30.77%)	9 (17.65%)	1 (4.35%)	
	> = 91	0 (0%)	1 (1.96%)	0 (0%)	
BMI	thin	3 (11.54%)	12 (23.53%)	1 (4.35%)	0.172
	normal	10 (38.46%)	18 (35.29%)	11 (47.83%)	
	overweight	9 (34.62%)	10 (19.61%)	9 (39.13%)	
	obese	4 (15.38%)	11 (21.57%)	2 (8.7%)	
Education Level	>12th grade of high school	20 (76.92%)	24 (47.06%)	8 (34.78%)	**0.008**
	≤12th grade of high school	6 (23.08%)	27 (52.94%)	15 (65.22%)	
Comorbidities					
Diabetes	no	23 (88.46%)	46 (90.2%)	20 (86.96%)	0.914
	yes	3 (11.54%)	5 (9.8%)	3 (13.04%)	
Ischemic Heart Disease	no	24 (92.31%)	42 (82.35%)	23 (100%)	0.066
	yes	2 (7.69%)	9 (17.65%)	0 (0%)	
High Cholesterol	no	22 (84.62%)	38 (74.51%)	20 (86.96%)	0.367
	yes	4 (15.38%)	13 (25.49%)	3 (13.04%)	
Hypertension	no	14 (53.85%)	30 (58.82%)	18 (78.26%)	0.171
	yes	12 (46.15%)	21 (41.18%)	5 (21.74%)	
Polypharmacy	no	3 (11.54%)	20 (39.22%)	19 (82.61%)	**< 0.001**
	yes	23 (88.46%)	31 (60.78%)	4 (17.39%)	
Current-smoking status	no	26 (100%)	48 (94.12%)	22 (95.65%)	0.458
	yes	0 (0%)	3 (5.88%)	1 (4.35%)	
Influenza vaccine	no	14 (53.85%)	26 (50.98%)	10 (43.48%)	0.754
	yes	12 (46.15%)	25 (49.02%)	13 (56.52%)	
Pneumococcal vaccine	no	26 (100%)	50 (98.04%)	22 (95.65%)	0.555
	yes	0 (0%)	1 (1.96%)	1 (4.35%)	
SGRQ-C	Low quality ≥ 25	26 (100%)	50 (98.04%)	5 (21.74%)	**< 0.001**
	High quality< 25	0 (0%)	1 (1.96%)	18 (78.26%)	
	Mean (SD)	81.34 (7.82)	49.76 (14.15)	18.26 (8.29)	**< 0.001**
CAT	Low health status ≥ 10	26 (100%)	38 (74.51%)	1 (4.35%)	**< 0.001**
	High health status < 10	0 (0%)	13 (25.49%)	22 (95.65%)	
	Mean (SD)	27.04 (6.2)	15.25 (6.79)	5.35 (2.77)	**< 0.001**
mMRC	High level of breathlessness ≥ 2	26 (100%)	51 (100%)	12 (52.17%)	**< 0.001**
	Low level of breathlessness < 2	0 (0%)	0 (0%)	11 (47.83%)	
	Median (25–75 percentile)	4 (3–4)	2 (1–2)	1 (0–1)	**< 0.001**
FEV1(%) mean(±SD)		39.5(±14.45)	49.65(±17.16)	59.78(±12.31)	**< 0.001**
Number of administered drugs Median (25–75 percentile)		6 (5–8)	6 (4–8)	3 (2–4)	**< 0.001**
Disease Duration (years) Median (25–75 percentile)		9 (4–17)	4 (1–10)	4 (1–8)	**0.04**

Assessment of the relation between independent variables and medication adherence showed that education, polypharmacy, SGRQ-C, CAT, mMRC, FEV_1_%, number of administered drugs, and years diagnosed with COPD were statistically significant. Cut-off point of education level was defined as high school diploma degree which was attributed to those graduated from 12th grade of high school. In this case, patients with high school diploma degree or upper had higher medication adherence (*p* = 0.008). Patients with polypharmacy tended to have lower medication adherence (*p* < 0.001). Participants with high quality of SGRQ-C, CAT and mMRC had higher medication adherence (*p* < 0.001). As it can be seen, as FEV_1_% increases medication adherence also increases (*p* < 0.001). However, with every unit of increase in number of administered drugs and years diagnosed with COPD, medication adherence decreases (*p* < 0.001 and 0.04, respectively) (Table 1).

In the next step, an ordinal logistic regression was performed on the so-called significant variables. In Table 2, the parameter estimates along with their confidence intervals and their *p*-values are indicated. Among these 8 covariates, education, polypharmacy, SGRQ-C and CAT were found to be significantly associated with medication adherence while controlling for other covariates. Variables which were not significantly associated with medication adherence i.e. FEV_1_%, number of administered drugs, years diagnosed with COPD and mMRC were excluded from the final model. It should be noted that non-significancy of mMRC may be due to zero frequency of two cells as presented in Table 1.

**Table. 2:** Primary ordinal logistic regression on significant variables and medication adherence

Variable	Parameter estimate (Exp(B))	95% Confidence Interval for Exp(B)		Wald Chi-Square	*P*-Value
		Lower	Upper		
FEV1 (%)	1.027	0.993	1.062	2.350	0.125
Number Of Administered Drugs	1.214	0.869	1.694	1.295	0.255
Education	4.257	1.462	12.397	7.055	**0.008**
Disease Duration (years)	0.967	0.909	1.027	1.195	0.274
Polypharmacy	0.080	0.012	0.530	6.860	**0.009**
SGRQ-C	13.561	1.017	180.796	3.892	**0.049**
CAT	32.140	3.401	303.737	9.169	**0.002**
mMRC	NA^a^	–	–	–	–

^a^ Not Applicable due to zero frequency in two categories of medication adherence as reported in Table 1

The final regression model is represented in Table 3. As can be seen, all 4 covariates have significant impact on medication adherence. It can be interpreted that the odds of higher medication adherence in patients with high school diploma or upper is 3.78 times greater than those with lower diploma education, while controlling for other 3 covariates (*p* = 0.01). Also, patients with high HRQoL and high health status have higher odds (SGRQ-C = 50.67 and CAT = 35.43) of high medication adherence while controlling for other covariates (*p* = 0.002 and *p* = 0.001, respectively). On the other hand, odds of patients with polypharmacy having high medication adherence is 81% less than those without polypharmacy, while controlling for other covariates (*p* = 0.008). Also, in the binary logistic regression analysis, the impact of medication adherence on HRQoL was not statistically significant. (*p* = 0.99).

**Table. 3:** Final ordinal logistic regression on significant variables in Table 2

Variable	Parameter estimate (Exp(B))	95% Confidence Interval for Exp(B)		Wald Chi-Square	*P*-Value
		Lower	Upper		
Education	3.779	1.375	10.384	6.646	**0.010**
Polypharmacy	0.191	0.056	0.652	6.991	**0.008**
SGRQ-C	50.675	4.422	580.730	9.952	**0.002**
CAT	35.430	3.965	316.618	10.193	**0.001**

## Discussion

In this study, we found that patients who have polypharmacy tend to have less medication adherence but those with higher education level, higher quality of life and higher health status have more tendency towards higher medication adherence.

Medication adherence in our study was measured by the Persian version of MMAS-8 as a trichotomous variable in which 74% of patients distributed in high and medium adherence categories. Several studies have examined adherence to medications in COPD patients with reports mentioning the rate of medication adherence between 22.3% to approximately 60% in real-world clinical practice [6, 14]. To assess the level of medication adherence, several methods have been developed which can be classified as subjective and objective [6]. Each method have different cut-points defining the patient is either adherent or non-adherent. The differences in study design (cross-sectional versus longitudinal), sample size, population, pharmacotherapy, and adherence measurement methods, could affect the results of medication adherence in these studies. These factors could be the reason why the proportion of adherent patients in our and some studies [15, 16] is higher. However, it has been found that among chronic diseases the medication adherence is specifically low in COPD patients [4].

Understanding associated factors with medication non-adherence could inspire the patients take the appropriate treatment. These factors include: socioeconomic factors, social/familial support, unemployment, low income, less education, living alone, comorbidities, smoking status, satisfaction with clinician expertise, and so on [6]. The so-called factors could be considered as a guidance for the government and health care providers in policy making strategies. However, in our study, no significant association was found between age, gender, comorbidities, BMI, current smoking status, influenza and Pneumococcal vaccine, with medication adherence, which is consistent with the results of the several studies in COPD patients [15, 17–21]. Nevertheless, there are lines of evidence which demonstrate that there is a significant correlation between age and medication adherence [8, 22]. In clinical practice, accessible interventions to improve adherence could be employed; including: providing organized and comprehensive disease-specific educations for patients, applying motivational interventions for patients and their caregivers, consultation with clinical pharmacists, enhancing healthcare providers’ communication skills and counseling, and using medication-taking reminders [5, 6]. Our study is a cross-sectional survey and evaluation of all of factors affecting adherence may not be possible. However, amongst the above factors, instructions on how to use inhaler devices were provided at each visit and patients were trained constantly and precisely. Positive effects of constant training of inhaler devices on medication adherence has been proved in some cohort studies containing COPD patients [23, 24], but we were not able to evaluate these effects due to limitations pertaining to our study design.

The impact of medication adherence on HRQoL was assessed in several studies, which could associate negatively [25] or positively [26] with quality of life, or there might be no association [27]. But, in our knowledge, the impact of HRQoL on medication adherence was assessed only in one study [8]. Ágh et al. found a negative relationship between HRQoL and medication adherence [8]. So that, better quality of life was associated with non-adherence. Whereas in our study, in spite of no impact of medication adherence on HRQoL which could be probably due to the snapshot nature of the cross-sectional studies, we found that HRQoL has a positive impact on adherence. Reasons to this contradiction may be due to the high proportion of our population with low HRQoL and high disease severity, and also our patients have lower mean FEV1% compared with Ágh et al., study [8] in non-adherent group (39.50 vs. 61.54, respectively) which all points to worse disease status of patients in our study.

Although, it is obvious that differences in study population and therapy along with diversity of methodologies have led to differences in the results of the studies around HRQoL; but, one of the most substantial differences is heterogeneity in tools used to measure quality of life. For instance, Ágh et al. applied EQ-5D to measure HRQoL in COPD patients [8] which is a general instrument and therefore may not be adequately sensitive to recognize the level of HRQoL in COPD patients [3]. While we incorporated SGRQ-C which is a COPD-specific instrument and is extensively applied in COPD setting as an appropriate tool to evaluate quality of life [25, 28]. However, to obtain a broad view of different aspects of HRQoL, it is recommended that generic (e.g. EQ-5D) and disease-specific (e.g. SGRQ-C) instruments to be used simultaneously [3].

Despite the issue of choosing appropriate instruments to measure disease-specific quality of life, it has been suggested that the relationship between medication adherence and HRQoL may be dual [3]. At the beginning of the treatment, medication adherence improves HRQoL by decreasing disease symptoms and improving health status. But, in the long-term, improvement in HRQoL may be considered a trigger for non-adherence [3, 8]. We could expect that if HRQoL in majority of the our population shifts towards a better level, which has a low chance due to progressive nature of COPD, it may cause the adherence to decrease in a long-term period. But, given that in our study the proportion of patients with low HRQoL is high, by increasing HRQoL we could foresee that medication adherence also increases. However, when the adherence starts to decrease is not pre-specified. Maybe taking into account the patients’ perception towards their disease and HRQoL status and when to stop their medication could help the researchers to identify this substantial time point.

To assess a comprehensive disease-specific health status, along with a spirometric measurement of FEV1%, patient-reported outcome instruments of disease severity e.g. CAT and/or mMRC should be used as well [11], since there are reports that indicate functional severity of the disease measured by FEV1% do not fully reflect health status in COPD patients [29, 30]. The literature review shows that there is no solidarity on which of FEV1%, CAT, and mMRC is better for assessing the relationship between severity of disease and medication adherence [8, 15, 25, 31–33]. In concordance with this, when FEV1%, CAT, mMRC were added to the regression model, Duarte-de-Araújo found the association between adherence and FEV1% to be more powerful than that between adherence and mMRC or CAT [15]. Whereas, we found this association more powerful for CAT rather than FEV1%. Regardless of this disagreement, it has been shown that patients with severe disease and lower degree of health status are [15, 34, 35] more likely to be adherent to treatment. However, in our study patients with better health status have more adherence to their medications. Some of the notable items that affect this inconsistency, which were not measured in our study, might be beliefs and perceptions of the patients regarding their disease and necessity of taking their medications [36, 37]. The adverse effects of non-adherence on the emergence of patients’ symptoms and health status are quite expected [31, 32, 38] and if the patient do not take the medications properly, disease severity and symptoms increase in short term which is easily realized by the patients [39]. Therefore, when patients believe that their health status is affected by the disease and adherence keeps them away from consequences of non-adherence, hence, in order to obtain the benefits of medication adherence i.e. high health status, they take their medication more willingly [37, 40]. Additionally, in our study on the contrary with Bosley et al., [26] who proposed that adherence is related to the current health status of the patient and is not resulting from previous experience of illness, we hypothesized that the patients’ persistency in maintaining their treatment may be due to their good experience in medication taking. In fact, as we mentioned above, medication adherence leads to positive effects of treatment and could promote the patients’ perception regarding the necessity of taking their medication. So, being at a good level of health status may lead to more adherence to medications. To conclude our findings, it seems we need to assess patient-related factors affecting medication effectiveness i.e., knowledge, attitudes, beliefs, perceptions and expectations, rather than socio-demographic factors or other clinical factors [7, 41].

Moreover, our observation provided evidence that literate people had more medication adherence. This can be due to the knowledge of educated people who understand the necessity of regular management of their disease, which leads to the proper adherence. In accordance with our findings, people who had high school or university education had better medication adherence [42]. Similarly in another study, higher education levels in veterans with COPD led to greater inhalants acceptance [43]. However, there are some reports about non-significant correlation between education and medication adherence [15, 17, 20, 21].

COPD patients with several comorbidities take six medications on average each day [4]. Adherence in COPD is associated with the number of medications the patient takes daily [44]. Polypharmacy is common in COPD patients with persistent signs. Vetrano et al. reported that polypharmacy caused non-adherence especially in COPD patients older than 65 [14]. Also, health status is a significant predictor of polypharmacy in addition to disease severity [45]. Ágh et al. showed that there was a negative association between the number of prescribed pulmonary medications and their daily dose with medication adherence [8]. In favor of these results, we also found that patients have better adherence when they have no polypharmacy, which is probably because of sensitivity to the drugs side effects, medication interference with daily schedules or forgetfulness.

### Limitations

Our study had some limitations. We measured the association between quality of life and medication adherence in a cross sectional time frame. It is suggested that in order to evaluate the factors affecting medication adherence (such as the role of training patients) and also the long-term effect of medication adherence on quality of life and vice versa, a longitudinal study should be conducted.

Another limitation we faced was small sample size. Wide confidence intervals of CAT and SGRQ-C in ordinal regression and non-significant mMRC may be due to the small sample size in our study. Also, other factors such as socio-economic status of patients might have influenced the results. However, it should be noted that in our study medication adherence was defined as a trichotomous variable, while in similar studies it was a dichotomous variable.

We applied the Persian version of the MMAS-8 questionnaire which was filled out in a supervised interview. Despite there are different tools to measure different characteristics of medication adherence, currently there is no consistency in a globally-approved tool. Thus, it is needed and recommended to have a universal agreement on a unique tool for this. Moreover, self-reported questionnaires might end in overestimating the effects of interest, therefore applying objective adherence measurements (i.e., dose counting, electronic medication monitoring systems, percentage of the total doses of medication taken, possession ratio) along with self-report questionnaires could improve the accuracy of detecting the true level of medication adherence [6].

Taking into account the fact that medication adherence is strongly influenced by patients’ perception of the disease and the clinician expertise and their insight about the necessity of the medication, persuades us to measure them properly.

Regarding psychiatric disorders are frequent among patients with chronic diseases [40], in COPD patients specifically, depression is more frequently seen in women rather than men [35]. Evaluation of this disorder and other psychiatric disorders on medication adherence would be of importance in COPD setting.

## Conclusions

In our study, adherence to medication is influenced by quality of life, health status, polypharmacy, and education. Patients’ good experience from previous positive effects of medication intake, which manifested in enhancement of quality of life and health status could encourage patients to continue medication consumption. Apart from the effective variables on adherence, in order to increase medication adherence while maintaining and/or increasing quality of life, patients’ view of the medication effectiveness should be taken into account. One should note that quality of life in COPD patients is a critical factor which should be considered as priority when trying to increase medication adherence.

## Data Availability

The datasets used and/or analysed during the current study are available from the corresponding author on reasonable request.

## Abbreviations

BMI: Body Mass Index
CAT: COPD Assessment Test
COPD: Chronic Obstructive Pulmonary Disease
FEV1: Forced Expiratory Volume in the First Second
HRQoL: Health-Related Quality of Life
MMAS-8-Item: The eight-item Morisky Medication Adherence Scale
mMRC: Modified British Medical Research Council
SGRQ-C: The St George’s Respiratory Questionnaire - COPD-Specific Version
